# A naturally evolved mutation (Ser59Gly) in glutamine synthetase confers glufosinate resistance in plants

**DOI:** 10.1093/jxb/erac008

**Published:** 2022-01-13

**Authors:** Chun Zhang, Qin Yu, Heping Han, Chaojie Yu, Alex Nyporko, Xingshan Tian, Hugh Beckie, Stephen Powles

**Affiliations:** 1 Guangdong Provincial Key Laboratory of High Technology for Plant Protection, Institute of Plant Protection, Guangdong Academy of Agricultural Sciences, Guangzhou, PR China; 2 Australian Herbicide Resistance Initiative, School of Agriculture and Environment, University of Western Australia, Crawley, WA, Australia; 3 Taras Shevchenko National University of Kyiv, Kiev, Ukraine; 4 INRAE-Bordeaux, France

**Keywords:** *Eleusine indica*, glufosinate, glutamine synthetase, target site mutation

## Abstract

Glufosinate is an important and widely used non-selective herbicide active on a wide range of plant species. Evolution of resistance to glufosinate in weedy plant species (including the global weed *Eleusine indica*) is underway. Here, we established the molecular basis of target site glufosinate resistance in *Eleusine indica*. Full-length *E. indica* glutamine synthetase (GS) iso-genes (*EiGS1-1*, *1-2*, *1-3*, and *EiGS2*) were cloned, and expression of *EiGS1-1* and *EiGS1-2* was higher than that of *EiGS2*. A novel point mutation resulting in a Ser59Gly substitution in EiGS1-1 was identified in glufosinate-resistant plants. Rice calli and seedlings transformed with the mutant *EiGS1-1* gene were resistant to glufosinate. Purified mutant EiGS1-1 expressed in yeast was more tolerant to glufosinate than the wild-type variant. These transgenic results correlate with a more glufosinate-resistant GS in the crude tissue extract of resistant versus susceptible *E. indica* plants. Structural modelling of the mutant EiGS1-1 revealed that Ser59 is not directly involved in glufosinate binding but is in contact with some important binding residues (e.g. Glu297) and especially with Asp56 that forms an intratoroidal contact interface. Importantly, the same Ser59Gly mutation was also found in geographically isolated glufosinate-resistant populations from Malaysia and China, suggesting parallel evolution of this resistance mutation.

## Teaser

This work reveals a naturally evolving point mutation in glutamine synthetase that confers resistance to glufosinate in the globally important C_4_weed species *Eleusine indica*.

## Introduction

Glufosinate (d,l-phosphinothricin) is a non-selective, broad-spectrum herbicide. Since first commercialized in 1993, glufosinate has become widely used for post-emergence weed control in global agricultural and non-agricultural land. With the introduction of transgenic glufosinate-resistant crops (maize, soybean, rice, canola, and cotton), glufosinate usage in the Americas has been increasing exponentially over the last decade. Glufosinate use will probably further increase as it replaces glyphosate (due to the widespread evolution of glyphosate-resistant weeds) and paraquat (which has been removed in some countries), and with future adoption of transgenic crops stacking multiple resistance traits, including glufosinate (Takano and Dayan, 2020).

Glufosinate is a competitive inhibitor of glutamine synthase (GS; EC 6.3.1.2) (Ray, 1989), a key enzyme involved in nitrogen metabolism by catalysing incorporation of ammonia into glutamate to form glutamine. Ammonia is produced from nitrate reduction, N_2_ fixation, photorespiration, and other plant cell processes (Leegood *et al.*, 1995). Photorespiration is probably the most important process that releases ammonium in the conversion of glycine to serine via the C_2_ cycle (Lea *et al.*, 1992; Leegood *et al.*, 1995). In plant species, there are two nuclear-encoded GS isoforms: cytoplasmic GS1 encoded by a small gene family, and plastidic GS2 encoded by a single gene. In leaf tissue of many C_3_ plants, GS2 is the predominant isoform, having much higher activity than GS1; in leaf tissue of C_4_ plants, the relative level of GS1 is either similar to, or even higher than, GS2 (Hirel and Gadal, 1982; McNally *et al.*, 1983).

Irreversible inhibition of GS by glufosinate results in high-level ammonia accumulation (Logusch *et al.*, 1991), causing inhibition of photosynthesis and leading to plant death (Manderscheid, 1993). However, this alone may not explain the contact activity of glufosinate causing rapid plant death. Recent studies suggest that the accumulation of reactive oxygen species (ROS) and subsequent lipid peroxidation triggers the quick action of glufosinate (Takano *et al.*, 2019, 2020; Takano and Dayan 2020).

Although glufosinate has been in commercial use for almost three decades, field resistance evolution has only been documented in a few weedy plant species including *Eleusine indica*, *Lolium perenne*, biennial *Lolium multiflorum* and annual *Lolium rigidum* (Jalaludin *et al.*, 2010; Avila-Garcia and Mallory-Smith 2011; Ghanizadeh *et al.*, 2015; Fernandez *et al.*, 2017; Travlos *et al.*, 2018). Given that field-evolved resistance to glufosinate is limited, few mechanistic studies have been conducted. Glufosinate resistance in one *L. multiflorum* population was found due to non-target site enhanced glufosinate metabolism (although initially incorrectly determined as due to an Asp183Asn mutation in the GS2 isoform) (Brunharo *et al.*, 2019). Target site GS mutations that endow glufosinate resistance (e.g. His249Tyr and Arg295Lys) were intentionally laboratory selected (such as by DNA shuffling or direct evolution) in soybean cell lines and rice (Pornprom *et al.*, 2008; Tian *et al.*, 2015), but no field-evolved target site resistance mutations have thus far been identified.

Glufosinate resistance was first reported in Malaysian *E. indica* populations (Chuah *et al.*, 2010; Jalaludin *et al.*, 2010), but the resistance mechanisms still remain elusive (Jalaludin *et al.*, 2017). Taking advantage of our in-house *E. indica* genome data, in this study we re-examine target site resistance mechanisms in the resistant Malaysian *E. indica* population (Jalaludin *et al.*, 2010) by *GS* iso-gene cloning and sequencing. We identify and functionally characterize a GS1 target site resistance mutation Ser59Gly. We explore the enzyme kinetic and structural basis of this GS1 mutation and discuss why this mutation can be selected in the field in a C_4_ plant species. In addition, we provide evidence that glufosinate resistance is emerging in geographically isolated *E. indica* populations from South China through parallel evolution of this same mutation.

## Materials and methods

### Plant materials and herbicide treatment

The glufosinate-resistant (R) *E. indica* subpopulation derived from a multiple herbicide resistance population and a glufosinate-susceptible (S) population (Jalaludin *et al.*, 2015) were used in this study. The R population was further purified by treating four-leaf stage plants with glufosinate (Basta, 200 g l^−1^, SC; Bayer CropScience) at 990 g ha^−1^ (2× recommended field rate). Glufosinate was applied using an in-house cabinet sprayer delivering 118 l ha^−1^ at 200 kPa with a speed of 1 m s^−1^. Plants were grown in a glasshouse during the summer season at the University of Western Australia (Perth, Australia). Surviving individuals (eight plants), together with eight untreated plants from the S population, were separately bulked up for seeds, and progeny plants were used for subsequent experiments.

In addition, *E. indica* seeds were collected from 18 field populations (exposed and random samples) from Guangdong province, South China (Table 1), as well as a known glufosinate-S population (referred to as S1) with no glufosinate exposure history. Seedlings were grown outdoors in pots contining autoclaved field soil during the summer growing season at the Academy of Guangdong Agricultural Sciences (Guangzhou, China). At the four- to five-leaf stage, seedlings (40 seedlings per population) were treated with glufosinate (990 g ha^–1^) in a laboratory sprayer delivering 270 ml min^−1^ at 0.3 MPa with a speed of 0.4 m s^−1^ (model ASS-4. Beijing, China). Plant survival was determined 3 weeks after treatment and the experiment was repeated.

**Table 1.** Presence and absence of the Ser59Gly mutation in surveyed field populations from South China

Population	Location	Resistance status	No. of plants analysed	No. of plants with genotype of EiGS1-1	
				Ser59 (WT)	Gly59 (mutant)
S1	Fallow field, Guangzhou, China (22°8ʹN, 113°44ʹE)	S	15	15	
P1	Baiyun, Guangzhou, China (23°35ʹN, 113°39ʹE)	R	18	6	12
P2	Panyu, Guangzhou, China (22°8ʹN, 113°44ʹE)	r	15	14	1
P3	Huadu, Guangzhou, China (23°48ʹN, 113°36ʹE)	r	18	17	1
P4	Sanshui, Foshan, China (23°48ʹN, 112°9ʹE)	r	15	14	1
P5	Yangcun, Huizhou, China (23°48ʹN, 114°46ʹE)	r	12	12	
P6	Jianggao, Guangzhou, China (23°33ʹN, 113°23ʹE)	S	9	9	
P7	Xiangang, Zhaoqing, China (23°04ʹN, 112°64ʹE)	S	10	10	
P8	Boluo, Huizhou, China (23°52ʹN, 114°56ʹE)	S	15	15	
P9	Maoming, China (21°68ʹN, 110°88ʹE)	S	8	8	
P10	Mazhang, Zhanjiang, China (21°27ʹN, 110°3ʹE)	S	6	6	
P11	Qigong, Yangshan, China (24°32ʹN, 112°63ʹE)	S	11	11	
P12	Jiutan, zengcheng, China (23°11ʹN, 113°58ʹE)	S	17	17	
P13	Cenxi, Guangxi, China (23°03ʹN, 111°03ʹE)	S	13	13	
P14	Yulin, Guangxi, China (22°43ʹN, 110°07ʹE)	S	8	8	
P15	Sanya, Hainan, China (23°42ʹN, 109°28ʹE)	S	14	14	
P16	Lingshui, Hainan, China (18°51ʹN, 110°04ʹE)	S	16	16	
P17	Wenchang, Hainan, China (19°61ʹN, 110°72ʹE)	S	11	11	
P18	Qionghai, Hainan, China (19°25ʹN, 110°47ʹE)	S	13	13	

R, r, and S refer to populations that had >50%, 1–10%, and 0% plant survivors, respectively, when treated with glufosinate at the rate (990 g ha^−1^) that fully controls the susceptible (S1) population. The r populations have zero to low frequency of the mutation, suggesting non-target site resistance mechanisms.

### *EiGS* iso-gene (cDNA) cloning, mutation identification, and expression analysis

Total RNA was extracted from *E. indica* leaf tissue using the ISOLATE II RNA Plant kit (Bioline). Genomic DNA was removed using the TURBO-DNA free kit (Ambion), and cDNA was synthesized using the SuperScript III reverse transcriptase kit (Invitrogen). Primers for cloning full-length coding sequences of *E. indica GS1* iso-genes (*EiGS1-1*, *1-2*, and *1-3*) and the *GS2* gene (*EiGS2*) (Table 2) were designed based on the published *E. indica* transcriptome (Zhang *et al.*, 2021) and in-house *E. indica* genome sequencing data. The PCR conditions were: 98 °C for 10 s, 35 cycles of 98 °C for 10 s, 58 °C for 15 s, 72 °C for 30 s, and a final extension at 72 °C for 10 min. Sequences of the *EiGS1* iso-genes and *EiGS2* from R and S *E. indica* were amplified and compared plants to identify mutations. For *EiGS* iso-gene expression analysis, R and S *E. indica* seedlings (at the five-leaf stage) were treated with 0 and 495 g ha^−1^ glufosinate, and total RNA was isolated from the third leaf (fully expanded) of each R and S seedling 24 h after treatment using an RNA extraction kit (Tiangen, Guangzhou, China). DNA contamination in the isolated RNA samples was removed with DNase I (2 U per 1.5 μg RNA sample). Reverse transcription of each RNA sample was performed using a Reverse Transcriptase M-MLV Kit (TransGen Biotech, Beijing, China). Real-time quantitative PCR (qPCR) was conducted using a SYBR Green kit (Aidlab, Beijing, China) and primers are listed in Table 2. A housekeeping gene, eukaryotic initiation factor 4A (*eIF-4*), was tested and selected for normalization of *EiGS* iso-gene expression (Chen *et al.*, 2017). Melting curve analysis revealed a single peak of each PCR product, indicating specificity of the primers used. The relative *EiGS* iso-gene expression level was expressed using the 2^−ΔCt^ method. There were three replicate samples per treatment per population, and each sample contained pooled leaf material from five plants.

**Table 2.** Primers used for glutamine synthetase (GS) gene cloning and expression analysis in *Eleusine indica*

Primers	Sequence (5ʹ–3ʹ)
**GS gene cDNA cloning**	
EiGS1-1-F	ATGGCCTCCCTCACCGACCTC
EiGS1-1-R	CACCACTAAATAGATGGACGAG
EiGS1-2-F	TGCTTTGGCTTGTGAGATATGG
EiGS1-2-R	TGCACAGATCGTCACTCCGGC
EiGS1-3-F	TGCGACGCACAATTCACTCGC
EiGS1-3-R	GCTCGCACAAACGCATACCC
EiGS2-F	AGGATGTGGGGCGCCAGGAG
EiGS2-R	AATCACAAGTTCCAGACCGACA
**GS gene expression analyses (RT-qPCR)**	
qGS1-1-F	AGTCATTCGGGCGTGACATT
qGS1-1-R	AATGTAGCGAGCAACCCACA
qGS1-2-F	GCCGACATCAACACCTTC
qGS1-2-R	GGTAATCAGTTTCCCTTCCA
qGS1-3-F	AGCCCAGCCACTCCAATG
qGS1-3-R	GCGAAGTCCCAATAATACAAA
qGS2-F	GGTGTGGTGCTTACCCTTGA
qGS2-R	TTCACGCATGGTCTTTGTGC
eIF-4-F (reference gene)	CCTACCAAAACGACCACTACGAC
eIF-4-R (reference gene)	ATCACCACCGACCTCCTTGCTC
**pOX-GS recombinant vector construction and detection**	
pOX-GS1-F	TGTTACTTCTGCAGGGTACCATGGCCTCCCTCACCGACCTC
pOX-GS1-R	CGGATCCATAACGCGTTCAAGGCTTCCAGATGATGGTGGTC
Hyg-F	GACCTGCCTGAAACCGAACTG
Hyg-R	CCCAAGCTGCATCATCGAAA
**Transgene copy detection**	
TansGS1-F	AGAACGGCAAGGGCTACTTC
TansGS1-R	CCAGATGATGGTGGTCTCGG
SPS-F	TTGCGCCTGAACGGATAT
SPS-R	CGGTTGATCTTTTCGGGATG
**Yeast positive cloning test**	
Forward primer	CGTAGAATTCATGCACCATCACCATCACCATGCCTCCCTCACC
Reverse primer	TGTCTAAGGCGAATTAATTCGCGGCCGCTCAAGGCTTCCAG

### Glufosinate resistance genotyping and phenotyping

A glyphosate-R subpopulation derived from the multiple resistant *E. indica* population (Jalaludin *et al.*, 2015) and possessing the target site 106 mutation in 5-enolpyruvylshikimate3-phosphate synthase (EPSPS) (Han *et al.*, 2017) was found segregating for glufosinate resistance. This subpopulation was used to examine the correlation between *EiGS* genotypes and glufosinate resistance/susceptibility phenotypes. Leaf material of five- to six-leaf stage R *E. indica* seedlings (together with the S population as control) were pre-harvested individually, snap-frozen in liquid nitrogen, and stored at –80 °C. Three days later, when leaf regrowth had occurred, the seedlings were foliar treated with 990 g ha^−1^ glufosinate. Glufosinate-R (survivors) and -S (killed) phenotypes were determined 3 weeks after treatment. RNA was prepared from pre-harvested samples and the relevant *EiGS1-1* gene amplified and sequenced using the primer pair EiGS1-1-F/EiGS1-1-R (Table 2).

### Rice genetic transformation and dose response to glufosinate

Wild-type and mutant *EiGS1* (*EiGS1-1*-*WT* and *EiGS1-1*-*R59*, respectively) coding sequences were amplified using the primer pair pOX-GS1-F and pOX-GS1-R (Table 2). The PCR conditions were 35 cycles at 98 °C for 10 s, 55 °C for 15 s, and 72 °C for 30 s, followed by 72 °C for 7 min. The amplicons were subcloned into a binary pOX (HA) vector under control of the ubiquitin promoter (Chu *et al.*, 2018) using the In-fusion HD Cloning kit (Takara). The recombinant vectors were introduced into *Agrobacterium tumefaciens* strain EHA105, which was then used to transform Nipponbare rice.

Hygromycin-resistant (50 mg l^−1^) and proliferating calli were transferred onto fresh N6D plates containing glufosinate at 0, 50, 100, 200, or 400 μM. For each glufosinate concentration, 10 transformed calli were used, and growth response to glufosinate was compared between WT and R59 transformants 18 d after treatment. Hygromycin-resistant and glufosinate-untreated calli were transferred to differentiation and rooting medium to obtain transgenic rice seedlings. The introduction of the transgene into rice calli and seedlings was confirmed by PCR using the primer pair HygF/HygR (Table 2) amplifying the hygromycin phosphotransferase (*hpt*) gene.

Seedlings of five T_1_ WT and five mutant R59 transgenic lines were tested for response to glufosinate (990 g ha^−1^). Surviving plants from each line were grown to maturity to obtain T_2_ seeds. The *EiGS1-1* gene copy number in T_2_ rice plants was estimated by qPCR (Ding *et al.*, 2004), with the sucrose phosphate synthase (*SPS*) gene used as the endogenous reference gene. Primers for *EiGS1-1* and *SPS* gene copy number detection are listed in Table 2. Three WT and three R59 T_2_ lines with a single copy of *EiGS1-1* were used for the glufosinate sensitivity test. These T_2_ lines were foliar sprayed with glufosinate (0, 248, 495, 990, 1480, and 1980 g ha^−1^ for the WT and 0, 495, 990, 1480, 1980, and 2970 g ha^−1^ for R59), and plant survival was determined 3 weeks after treatment. The experiment was conducted in a glasshouse during the normal warm rice-growing season at Guangdong Academy of Agricultural Sciences, China. There were three replicate pots per treatment each containing eight seedlings.

### Yeast transformation and recombinant EiGS1-1 protein purification

The *Eco*RI and *Not*I tags were created on the 5ʹ and 3ʹ ends of the coding region of *EiGS1-1-WT* and *EiGS1-1-R59* cDNA, which was fused with the His_6_ tag at the C-terminal end. The amplified PCR products were subcloned into the pPic9k vector and sequenced. The correct pPic9k-EiGS1-1-WT and pPic9k-EiGS1-1-R59 plasmids were linearized and transformed into *Pichia pastoris* strain GS115 competent cells, which were grown on BMGY medium plates. Positive clones were detected by PCR using the primer pair given in Table 2. Clones with high positive expression were selected and identified by SDS-PAGE. His-tagged protein in transgenic yeast was affinity purified using a nickel-nitrilotriacetic acid agarose column (Qiagen). The protein peak fractions were analysed for the presence of a single band of recombinant EiGS1-1 protein (~40 kDa) on the polyacrylamide gel and stained with Coomassie Blue (Supplementary Fig. S1).

### GS *in vitro* activity, glufosinate inhibition, and kinetics

GS activity in *E. indica* tissue extracts and in yeast recombinant EiGS1-1 proteins was measured by the γ-transferase assay (GS-dependent formation of γ-glutamyl hydroxamate) (Pateman, 1969) using a commercial detection kit (Solabio, Beijing, China). Assays were conducted in the presence of glufosinate at final concentration range of 0 to 10 mM, with small intervals between 0.01 mM and 1 mM. The reaction product was measured spectrophotometrically for absorbance at 540 nm. Protein concentration was determined for sample calibration. Assays using *E. indica* tissue extracts contained three biological replicates and those using yeast recombinant EiGS1-1 proteins contained three technical replicates.

Kinetic characterization (*K*_m_ and *V*_max_) of recombinant EiGS1-1 proteins was performed for the two substrates, glutamate and ATP, by the biosynthetic assay (Jalaludin *et al.*, 2017). The reaction mixture consisted of 100 mM Tris–HCl (pH 7.5), 10 mM ATP, 20 mM MgCl_2_, 30 mM hydroxylamine, and 20 mM glutamate.

### EiGS1 structural modelling

The spatial structure of the monomer and decamer of WT and mutant Ser59Gly isoforms of EiGS1 were reconstructed via homology modelling (Venselaar *et al.*, 2010), using the SwissModel web service (Waterhouse *et al.*, 2018). Search and scoring of structural templates were performed via internal tools of SwissModel. The spatial structure of the GS1 decamer from maize (*Zea mays*) deposited in the Protein Data Bank (Berman *et al.*, 2000) (accession no. 2D3C, Unno *et al.*, 2006) was used as a template for reconstruction. The identity level between the target and template amino acid sequences was found to be 93%. The spatial locations of ATP and glufosinate (phosphinothricin) molecules in appropriate binding sites on the GS1 surface were defined from positions of their structural analogues (ADP and phosphinothricin phosphate, respectively) in template spatial structure, and the location of glutamate (substrate) was defined from the position of its structural analogue, methionine-*S*-sulfoximine phosphate, in complex with maize GS1 (PDB accession code 2D3A, Unno *et al.*, 2006). Topologies of ATP, glutamate, and glufosinate for application in calculations of energy minimization and molecular dynamics simulations were performed via the web-based tool SwissParam (Zoete *et al.*, 2011). The position of the ammonium ion in the studied complexes was defined by its coordination between carboxyl groups of Glu297 and Asp56 known as binding residues (Unno *et al.*, 2006), and the carboxyl group of glutamate in the enzyme active site. The spatial geometry of the obtained complexes of the WT and mutant GS1 isoforms with ATP/glutamate and ATP/glufosinate were optimized via energy minimization using the L-BFGS algorithm (Das *et al.*, 2003) and Charmm27 force field (MacKerell *et al.*, 2000, 2004). Position-restrained molecular dynamics for canonical NVT (N for particle number, V for volume, T for temperature) and isothermal–isobaric NPT (P for pressure) ensembles within 100 ps intervals (to achieve the equilibrate state), and the unrestrained (productive) molecular dynamics of all studied complexes within 100 ns time intervals at 300 K were calculated in realistic intracellular conditions with the GROMACS software (Abraham *et al.*, 2015) version 2020. Computational details correspond to a procedure described in our previous work (Chu *et al.*, 2018). Data visualization was performed using Discovery Studio Vizualizer v 19.0.18287 (https://www.3ds.com/products-services/biovia/products/molecular-modeling-simulation/biovia-discovery-studio/).

### Statistical analysis

Glufosinate concentration causing 50% inhibition of GS activity (I_50_) or 50% plant mortality (LD_50_) was estimated by fitting the dose response data to a non-linear regression (four-parameter logistic) model, *y*=*c*+(D–C)/[1+(X/X_0_)^b^], where C is the lower limit representing plant survival or GS activity at infinitely large herbicide doses, D is the upper limit representing plant survival or GS activity at low herbicide doses close to untreated controls, X_0_ is the rate giving 50% response (LD_50_, I_50_), and b is the slope around X_0_. GS *K*_m_ and *V*_max_ values were estimated by fitting the data to the ligand binding model using SigmaPlot (version 12.0; SPSS Inc., Chicago, IL, USA). Significance of the difference in I_50_ and LD_50_ values between populations/lines was analysed by *t*-tests conducted using Prism 5.0 (GraphPad Software, La Jolla, CA, USA). Significance of the difference in sample means between treatments (e.g. gene expression and enzyme activity) was analysed by *t*-tests (*P*<0.05 or 0.01) using SPSS 18.0 (SPSS Inc.).

## Results

### Identification of the Ser59Gly mutation in EiGS1-1 of glufosinate-resistant *E. indica* plants

**Fig. 1.**
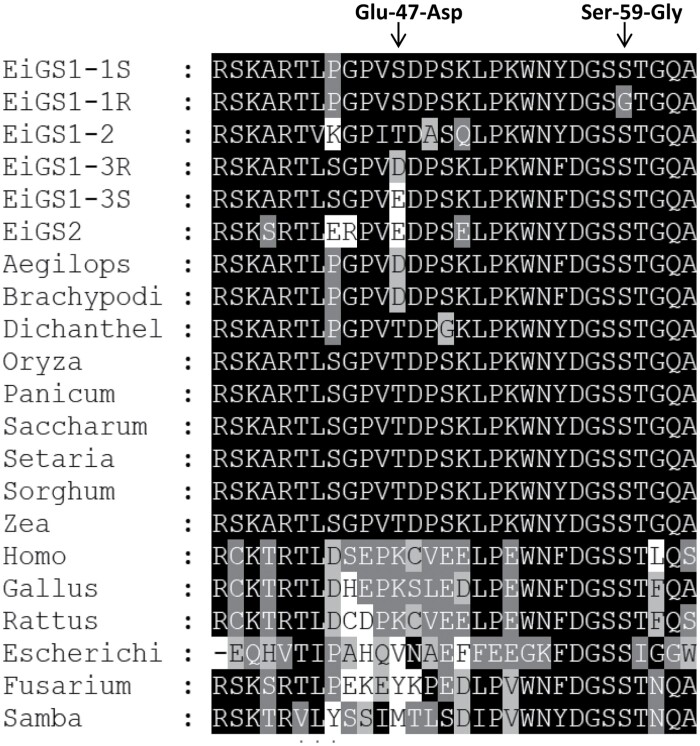
Partial sequence alignment of glutamine synthetase (GS1) genes of *E. indica* and other organisms near the Gly47Asp (EiGS1-3) and Ser59Gly (EiGS1-1) mutation sites (arrowed). Aegilops (*Aegilops tauschii*, LOC109764334), Brachypodi (*Brachypodium distachyon*, LOC100837122), Dichanthelium (*Dichanthelium oligosanthes*, OEL18127.1), Oryza (*Oryza sativa*, XP_015626102.1), Panicum (*Panicum miliaceum*, RLN07659.1), Saccharum (*Saccharum officinarum*, AAW21273.1), Setaria (*Setaria italica*, XP_0049538422), Sorghum (*Sorghum bicolor*, XP_021313946.1), Zea (*Zea mays*, NP_001105296.1), Homo (*Homo sapiens*, S70290.1), Gallus (*Gallus gallus*, M29076.1), Rattus (*Rattus norvegicus*, M91652.1), Escherichi (*Escherichia coli*, BAE77439.1), Fusarium (*Fusarium oxysporum*, KNB02176.1), Samba (Samba virus, AMK61947.1).

Full-length coding sequences of *E. indica* GS iso-genes (*EiGS1-1*, *EiGS1-2*, *EiGS1-3*, and *EiGS2*) (GeneBank accession numbers MZ888499, MZ888500, MZ888501, and MZ888502, respectively) were cloned from Malaysian glufosinate-R and -S populations. Comparison of the GS sequence between eight R and 15 bulked S samples revealed two single nucleotide polymorphisms (SNPs) resulting in two amino acid substitutions in R samples: a single nucleotide mutation of AGC to GGC, leading to a Ser59Gly substitution in EiGS1-1; and a mutation of GAA to GAT, leading to a Glu47Asp substitution in EiGS1-3 (Fig. 1). The identified R individuals were all found to be homozygous for the Ser59Gly mutation and the Glu47Asp substitution. As the Ser59 position is highly conserved in all GS isoforms in microorganisms, plants, and animals, whereas the amino acid equivalent to Asp47 occurs in many other plant species, we focused on the Ser59Gly mutation in the following experiments.

### Correlation of the Ser59Gly mutation with glufosinate resistance

Seedlings from within a single glyphosate-R population but segregating for glufosinate resistance were treated with 990 g ha^−1^ glufosinate, and 105 plants were identified as R and 15 as S phenotypes. The *EiGS1-1* gene was sequenced from 30 randomly selected R plants and all 15 S plants. The Ser59Gly mutation occurred in all 30 R plants, but in none of the 15 S plants. These results indicate that the Ser59Gly mutation is likely to be correlated with glufosinate resistance.

### Higher expression of the *EiGS1-1* iso-genes than of *EiGS2*

**Fig. 2.**
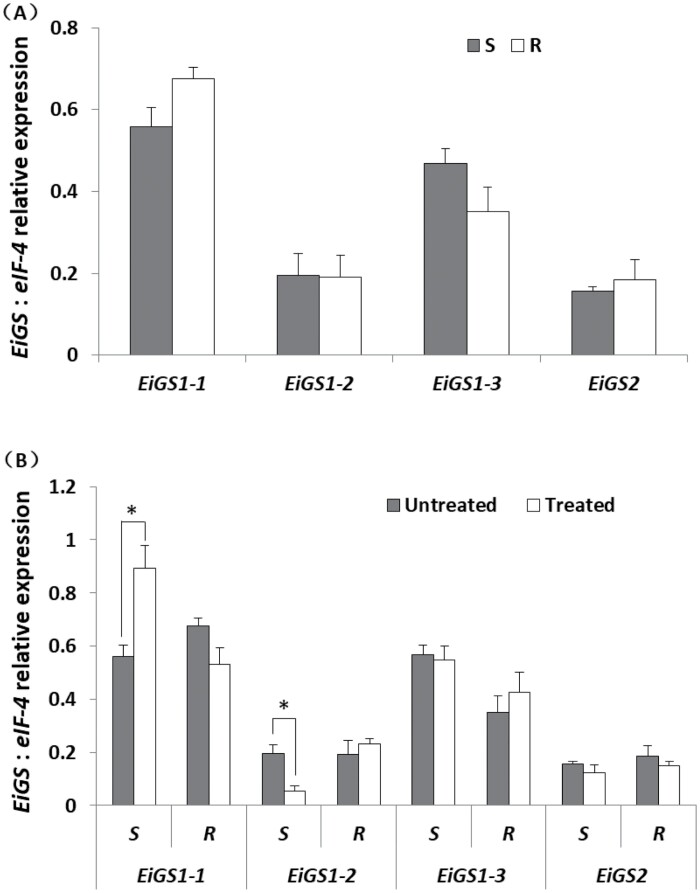
RT–qPCR analyses of *EiGS* iso-gene expression in leaf tissue of glufosinate-susceptible (S) and -resistant (R) *E. indica* (at the five-leaf stage). (A) Basal *EiGS* expression levels and (B) levels 24 h after glufosinate treatment (495 g ha^−1^). Data are means ±SE of three replicate samples each containing leaf material of five individual plants (∗ indicates significant difference according to the *t*-test, *P*<0.05).

In leaf tissue of *E. indica* (at the five-leaf stage), basal expression of *EiGS1-1* was the highest, followed sequentially by *EiGS1-3*, *EiGS1-2*, and *EiGS2* (Fig. 2). There was no significant difference in the expression of each iso-gene between R and S plants, although *EiGS1-1* tended to be higher and *EiGS1-3* lower in R versus S plants (Fig. 2A). Significant changes in *EiGS* iso-gene expression were not detected in response to glufosinate treatment, except for up- and down-regulation of *EiGS1-1* and *EiGS1-2*, respectively, in S plants (Fig. 2B).

### Glufosinate resistance of transgenic rice expressing the mutant *EiGS1-1* gene

**Fig. 3.**
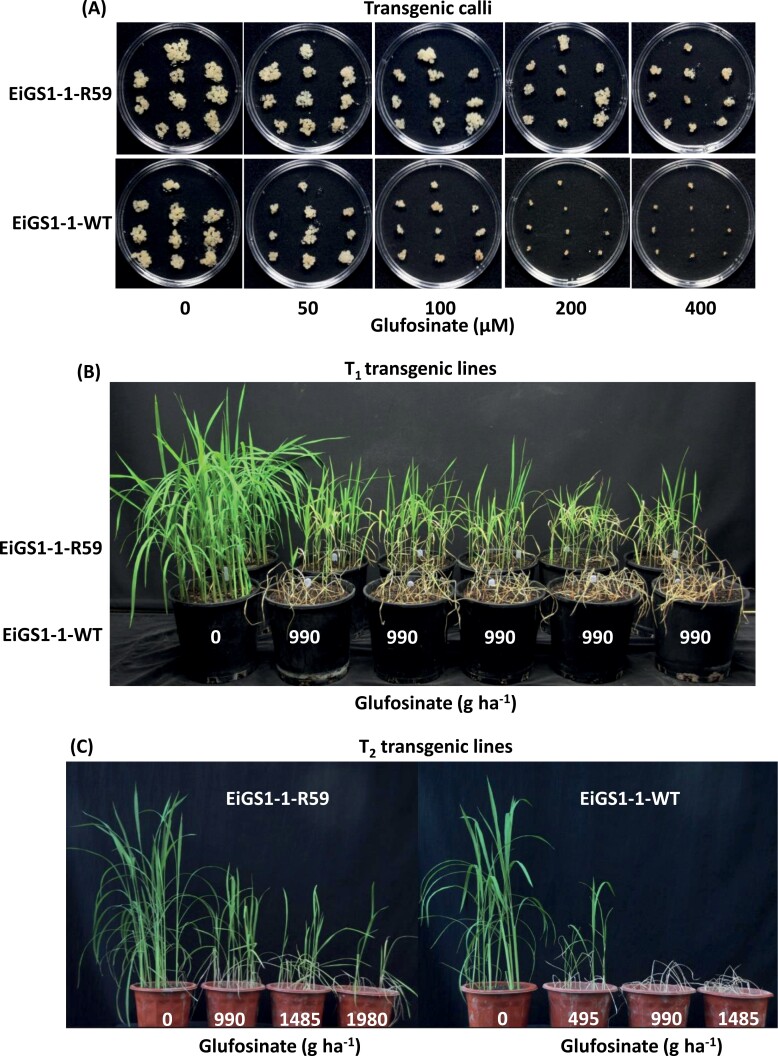
Expression of the *EiGS1-1-R59* gene confers resistance to glufosinate in transgenic rice calli (A), T_1_ (B, five lines), and T_2_ (C) seedlings, relative to rice calli/seedings expressing the *EiGS1-1-WT* gene. Images were taken 3 weeks after treatment.

Proliferation of *EiGS1-1-WT* rice calli was visibly inhibited by 50 μM glufosinate and totally arrested at 200 μM. In contrast, *EiGS1-1-R59* rice calli were less sensitive to glufosinate, such that continued growth occurred at 400 μM glufosinate (Fig. 3A).

**Fig. 4.**
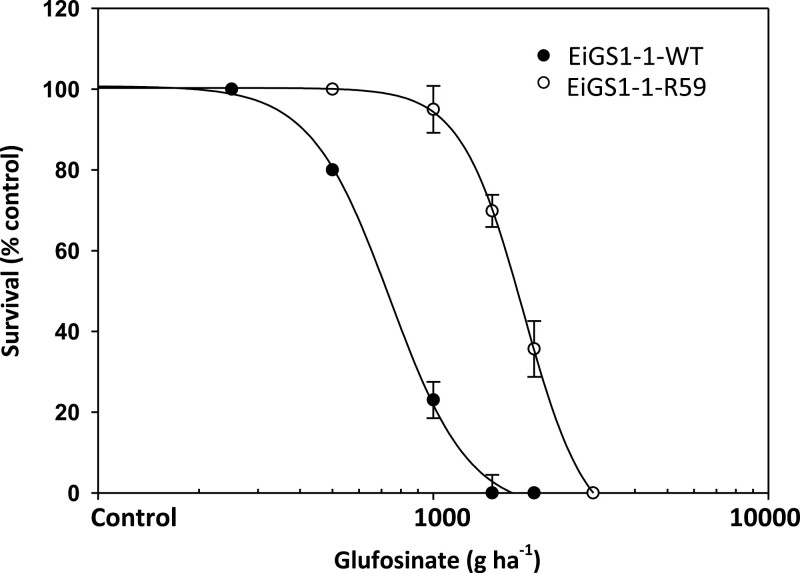
Glufosinate dose response of transgenic rice T_2_ seedlings expressing a single copy of *EiGS1-1-R59* versus *EiGS1-1-WT*. Plants were treated at the three- to four-leaf stage, and survival rate was determined 3 weeks after treatment. Data are means ±SE of pooled results of three WT and three R59 lines.

Seedlings of five T_1_ rice lines expressing *EiGS1-1-R59* mostly survived 990 g ha^−1^ glufosinate, whereas those of *EiGS1-1-WT* lines were killed by this rate (Fig. 3B). Three T_2_*EiGS1-1-R59* and three *EiGS1-1-WT* lines with a single transgene copy were evaluated for dose response to glufosinate. As expected, glufosinate at 990 g ha^−1^ killed the WT lines whereas R59 lines survived at higher rates (Figs 3C, 4; Table 3). Based on the LD_50_ R59/WT ratio, *EiGS1-1-R59*-expressing rice plants are 2.5-fold more resistant to glufosinate. These results further demonstrate that the Ser59Gly mutation in the *EiGS1-1* gene endows resistance to glufosinate, enabling plant survival at and above field glufosinate rates.

**Table 3.** Parameter estimates for logistic analysis of glufosinate dose required to cause 50% inhibition of plant mortality (LD_50_) for T_2_ transgenic rice lines expressing the wild-type (WT) and mutant (R59) *EiGS1-1* gene

Line	*C*	*D*	*b*	LD_50_ (g ha^–1^)	*P*-value (LD_50_)	I_50_ ratio (mutant/WT)
EiGS1-1-WT	−4.62 ± 3.3	101 ± 2.2	−3.66 ± 0.41	742 ± 32	*P*<0.01	/
EiGS1-1-R59	−10.86 ± 1.5	100 ± 0.43	−4.6 ± 0.41	1859 ± 16	*P*<0.001	2.5

The LD_50_ values estimated for WT and R59 lines are significantly different according to the *t*-test, *P*<0.0001.

### Reduced glufosinate sensitivity and enhanced activity of the mutant EiGS enzyme

#### Yeast recombinant EiGS1-1

**Fig. 5.**
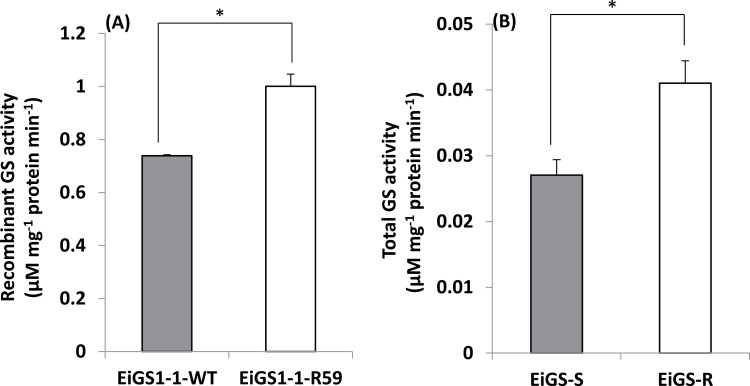
Glutamine synthetase (GS) activity of (A) purified yeast recombinant wild-type (WT) and mutant (R59) EiGS1-1 proteins and (B) in leaf extracts of glufosinate-susceptible (EiGS-S) and -resistant (EiGS-R) *E. indica* plants. Data are means ±SE of three biological replicates per treatment for EiGS-S and EiGS-R samples, and three technical replicates for yeast recombinant EiGS1-1 samples (∗ indicates significant difference according to the *t*-test, *P*<0.05).

**Fig. 6.**
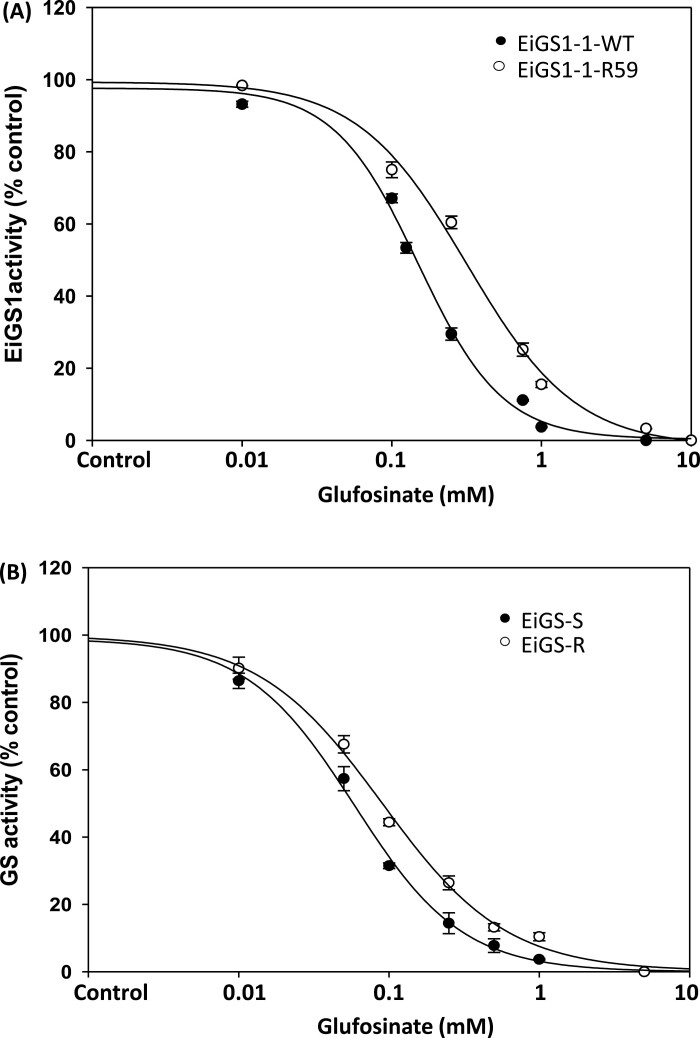
Glufosinate *in vitro* inhibition of glutamine synthetase (GS) activity of (A) purified yeast recombinant EiGS1-1-WT and EiGS1-1-R59 proteins and (B) in leaf extracts of glufosinate-susceptible (EiGS-S) and -resistant (EiGS-R) *E. indica*. Data are means ±SE of three biological replicates per treatment for EiGS-S and EiGS-R samples, and three technical replicates for yeast recombinant EiGS1-1 samples.

*EiGS1-1-WT* and *EiGS1-1-R59* were heterologously expressed and purified from yeast. The EiGS1-1-R59 variant showed 35% higher activity than EiGS1-1-WT in the absence of glufosinate (Fig. 5A). In the presence of glufosinate, EiGS1-1-R59 activity was significantly less inhibited than that of EiGS1-1-WT (Fig. 6A; Table 4). Based on the I_50_ R59/WT ratio, EiGS1-1-R59 is 2.3-fold more resistant to glufosinate than is EiGS1-1-WT (Table 4).

**Table 4.** Parameter estimates for logistic analysis of glufosinate dose required to cause 50% inhibition of GS activity in susceptible (S) and resistant (R) *E. indica* and in purified yeast recombinant wild-type (WT) and mutant (R59) EiGS1-1 protein.

Sample		*C*	*D*	*b*	I_50_ (mM)	*P*-value (I_50_)	I_50_ ratio (R59/WT, R/S)
Yeast recombinant protein	EiGS1-1-WT	0.28 ± 0.27	98 ± 0.18	−1.43 ± 0.02	0.15 ± 0.001	*P*<0.0001	/
	EiGS1-1-R59	−1.5 ± 0.29	98 ± 0.16	−1.39 ± 0.02	0.35 ± 0.003	*P*<0.0001	2.3
Leaf extract of *E. indica*	EiGS-S	0.007 ± 1.97	99 ± 2.25	−1.21 ± 0.12	0.059 ± 0.005	*P*<0.001	/
	EiGS-R	0.17 ± 2.7	100 ± 2.34	−1.05 0.11	0.09 ± 0.009	*P*<0.001	1.5

The I_50_ values estimated for WT and R59 lines, and S and R populations are significantly different according to the *t*-test, *P*<0.0001.

**Fig. 7.**
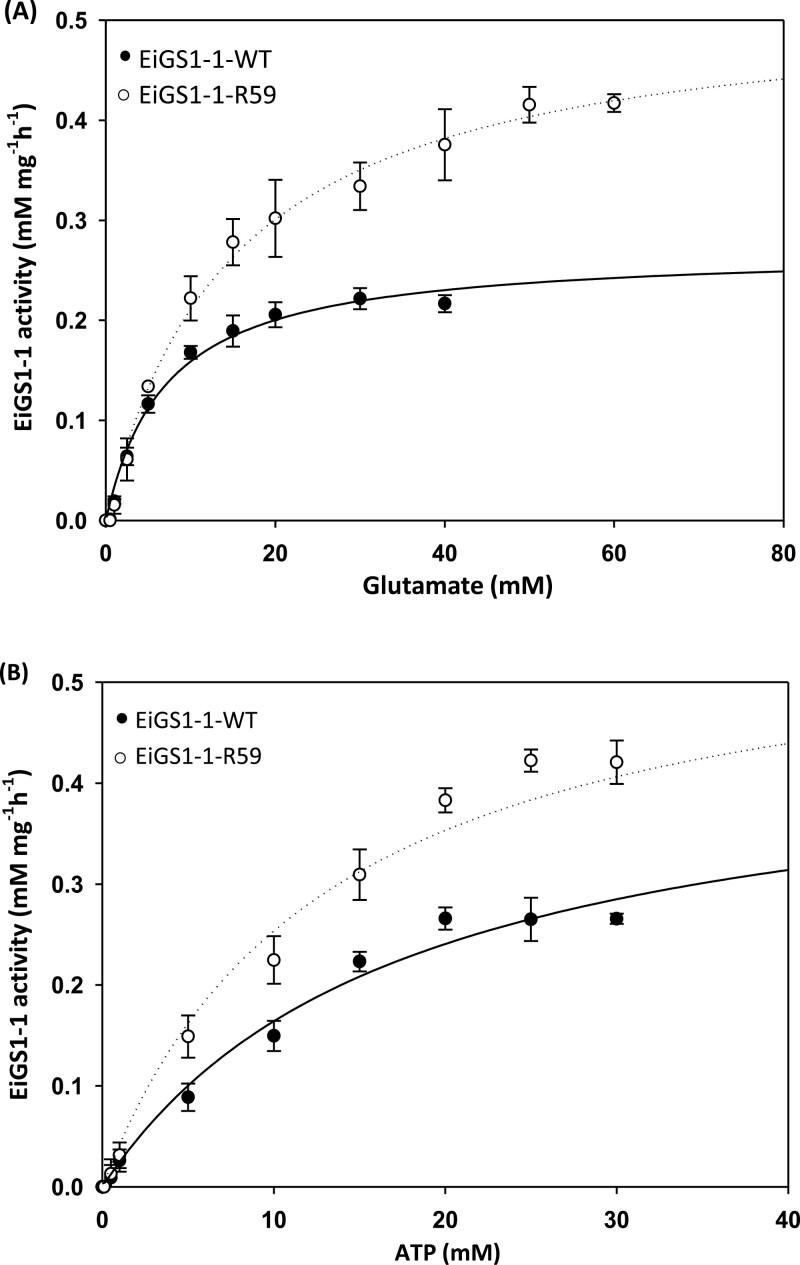
Enzymatic reaction kinetics of purified yeast recombinant EiGS1-1-WT and EiGS1-1-R59 proteins for the substrate glutamate and ATP. Data are means ±SE of three technical replicates of each sample.

Kinetic studies of the yeast recombinant proteins revealed that the Ser59Gly mutation slightly (up to 2-fold) increased the *K*_m_ and *V*_max_ for both glutamate and ATP, but had little impact on GS catalytic efficiency (*V*_max_/*K*_m_) (Fig. 7; Table 5).

**Table 5.** Kinetic parameters of the purified yeast recombinant wild-type (WT) and mutant (R59) EiGS1-1 proteins

GS variants	Substrate	Kinetic parameters		*V*_max_/*K*_m_
		*K*_m_ (mM)	*V*_max_ (mM mg^−1^ h^−1^)	
EiGS1-1-WT	Glutamate	6.9 ± 0.08 b	0.27 ± 0.004 b	0.035
EiGS1-1-R59		14.7 ± 0.09 a	0.52 ± 0.001 a	0.035
EiGS1-1-WT	ATP	17.4 ± 0.74 b	0.45 ± 0.007 b	0.026
EiGS1-1-R59		21.3 ± 0.27 a	0.75 ± 0.006 a	0.035

Data are means ±SE of three technical replicates of each sample, and the assay was repeated with similar results. Different letters in a column between WT and R59 genotypes indicate significant differences according to the *t*-test, *P*<0.01.

#### Total GS activity from R and S *E. indica*

Total GS activity in the crude extract of R plants was slightly (1.5-fold) but significantly higher than that of the S plants (Fig. 5B), probably due to the Ser59Gly mutation in GS1. GS from R plants was less sensitive (1.5-fold) to glufosinate than that from S plants (Fig. 6B; Table 4).

Given the consistency of LD_50_ and I_50_ ratios obtained from transgenic rice, yeast, and *E. indica* plants, it is clear that the Ser59Gly mutation in *EiGS1-1* reduces sensitivity to glufosinate and thus confers resistance.

### Presence of the Ser59Gly mutation in other glufosinate-R populations

Among 18 *E. indica* populations surveyed from South China, one population (P1) had >50% plant survivors at 990 g ha^−1^glufosinate (rated R), and four populations (P2–P5) had <10% survivors (rated r), while the remaining 13 populations had no survivors (rated S) (Table 1). The *EiGS1-1* gene was sequenced in up to 18 individuals from each population (including the survivors from R and r populations). The Ser59Gly mutation was only detected in the R and r populations (with a higher frequency in R than in r), and not in the 13 S populations.

### The Ser59Gly mutation indirectly affects glufosinate binding

GS structural modelling revealed that the binding site of the substrate glutamate is located on the GS1 surface near the intratoroidal contact interface (Supplementary Fig. S2A). This includes residues Glu131, Glu192, Val193, Gln197, Asn244, Gly245, Ala246, Gly247, His249, Agr291, His296, Glu297, Thr298, Arg311, and Arg332. Three of these (Val193, Glu297, and Arg311) are also immediate components of the appropriate contact interface. The glufosinate molecule shares the same binding site as glutamate. Thus, glufosinate inhibition of GS1 activity is competitive. However, glufosinate binds to more amino acids than glutamate (Supplementary Fig. S2B) and forms an attractive charge interaction with Glu297. Additionally, glufosinate has Van der Waals contacts with Tyr158 and Asp56 of the neighbouring subunit that is involved in forming the intratoroidal contact interface.

Being part of the contact interface from the side of the neighbouring subunit, Ser59 does not interact with glutamate or glufosinate directly, but contacts with residues Tyr158, Val193, Glu297, and Asp56 (two favourable H-bonds), and an ammonium ion (unfavourable donor–donor interaction, Supplementary Fig. S3A). The Ser59Gly substitution induces the structural rearrangement of the glutamate/glufosinate-binding site, including loss of contact with Val193 and NH_4_^+^ (Supplementary Fig. S3B), and thus, in turn, stabilizes the binding of the natural substrate glutamate with free binding energy reduction by 261 kJ mol^−1^. Consequently, this allows glutamate to successfully compete with glufosinate at the binding site, thus endowing resistance. Experiments examining the competitivity of glufosinate inhibition toward glutamate in mutant versus WT EiGS1-1 may comfirm this prediction.

It was observed that motif DGSS (Asp56, Gly57, Ser58, and Ser59) in *E. indica* GS1 is exceptionally conserved in GS of all eukaryotes and the majority of prokaryotes (e.g. bacteria and viruses) (Fig. 1). Specific amino acids in these positions are probably important for the stability of the intersubunit contact interface and entrance of the substrate glutamate into the active site..

## Discussion

In this study with glufosinate-R *E. indica*, a point mutation (Ser59Gly) in GS1 was identified and demonstrated to endow glufosinate resistance. Our early study (Jalaludin *et al.*, 2017) with the original glufosinate-R *E. indica* population failed to reveal this target site resistance. This is likely to be because mild changes in EiGS1-1 sensitivity conferred by the Ser59Gly mutation were difficult to detect in the crude enzyme extracts due to interference of GS2 and other GS1 isoforms in the assay, as well as some technical constraints, such as using large glufosinate concentration intervals (e.g. 10-fold increase). To overcome these limitations in the present study, we employed transgenic approaches including rice calli and seedlings for phenotypic resistance bioassays, and purified yeast recombinant EiGS1-1 mutant and WT variants for *in vitro* GS assays. We also used a further purified R population and optimized experimental conditions (e.g. glufosinate concentrations) in the GS assays. Consistent results were obtained from transgenic studies and from R and S *E. indica* plants.

Unlike C_3_ plants where the plastidic GS2 isoform is more abundant than the cytosolic GS1 isoforms, in C_4_ plants GS1 can account for >50% of total GS activity. For instance, in *Sorghum vulgare*, GS1 and GS2 represented 67% and 33% of total activity, respectively, in green leaves (Hirel and Gadal, 1982). In maize leaf tissue, GS1 had 61% activity in mesophyll cells and GS2 67% activity in bundle sheath cells (Gonzalez-Moro *et al.*, 2000). Indeed, as a C_4_ species, *E. indica* had higher leaf tissue expression of *EiGS1* (especially *EiGS1-1* and *EiGS1-2*) than of *EiGS2* (Fig. 2), which may correspond to their activity proportions, assuming that the transcript abundance, protein level, and enzyme activity of GS are related to each other.

There are several processes generating ammonium (NH_4_^+^) in the leaf, including photorespiration, nitrate/nitrite reduction, lignin biosynthesis, and protein turnover (Leegood *et al.*, 1995). Among these processes, photorespiration is probably the largest source of liberated NH_4_^+^, as photorespiratory NH_4_^+^ production may occur at rates up to 10 times that of nitrate/nitrite reduction (Leegood *et al.*, 1995). It is believed that plastidic GS2 plays a major role in recycling of NH_4_^+^ released from photorespiration. However, photorespiration is minimal in C_4_ plants compared with C_3_ plants, as C_4_ plants can concentrate CO_2_ at Rubisco in bundle sheath cells. Furthermore, GS1 in C_4_ plants is more sensitive to glufosinate inhibition (Gonzalez-Moro *et al.*, 2000) and more heat stable than GS2 (Hirel and Gadal. 1982).

Taken together, it seems that in C_4_ plants, GS1 may be an evolutionary hotspot for mutations that endow glufosinate resistance. Among other factors listed above, the gene expression level itself can be a genome constraint and higher expression can be the driving force for evolution of target site resistance (Tanigaki *et al.*, 2021).

It is perhaps not surprising that the field-evolved glufosinate resistance mutation in *E. indica* was identified at the Ser59 position of GS1. Firstly, the Ser59 residue within the motif DGSS is highly conserved in GSs of microorganisms, plants, animals, and humans (Fig. 1), indicating its importance for GS function. Secondly, as glufosinate is a competitive inhibitor of GS with respect to glutamate (Manderscheid and Wild, 1986; Unno *et al.*, 2006), mutations occurring directly in the binding site would confer higher levels of resistance, but may also incur a fitness cost (by lowering substrate affinity). GS is a highly allosteric enzyme, so even mutations outside the active site could exert an impact on herbicide binding, and thus contribute to resistance. Spatial reconstruction of *E. indica* GS1 complexes indicated the structural basis of glufosinate resistance endowed by the Ser59Gly mutation is an indirect effect via interactions with amino acids (e.g. Val193, Glu297, or Asp56) involved in glufosinate binding and intratoroidal contacts (Supplementary Fig. S2B). This type of mutation(s), although usually conferring low-level resistance, may have evolutionary advantages by reducing the resistance fitness cost. GS *in vitro* inhibition assays showed that the Ser59Gly mutation slightly reduced GS1 sensitivity to glufosinate, whereas rice transgenics demonstrated that this mild change in GS1 sensitivity is sufficient to endow resistance to the recommended field and higher rates of glufosinate (495–990 g ha^−1^) (Fig. 4). GS kinetics studies revealed that the Ser59Gly mutation had little adverse impact on catalytic efficiency of EiGS1-1 (Table 5). These features may, in part, explain why selection of the Ser59Gly mutation is favoured in the field. This is also true for the target site 106 mutation being away from the glyphosate EPSPS binding site, which endows resistance to glyphosate without a major fitness penalty (Han *et al.*, 2017).

We found that the Ser59Gly mutation occurs not only in the Malaysian glufosinate-R *E. indica* population, but also in glufosinate-R populations from China (Table 1), indicating independent parallel evolution. With increased glufosinate selection pressure, this relatively weak mutation may be replaced by other stronger single mutations, or even multiple mutations, such as the laboratory-generated *GS* mutations (Pornprom *et al.*, 2008; Tian *et al.*, 2015) and the naturally occurring glyphosate resistance double and triple *EPSPS* mutations (Yu *et al.*, 2015; Perotti *et al.*, 2019).

It was observed that only a homologous Ser59Gly mutation was present in all R and r *E. indica* populations examined, which may be due to high-level self-pollination, or alternatively may imply a recessive nature for resistance with respect to recommended or higher herbicide field application rates. A single resistance allele may be insufficient in endowing resistance to herbicides targeting multiple copies of a protein in diploid and polyploid species, especially for weak target site mutations, as has been demonstrated for acetyl co-enzyme A carboxylase (*ACCase*), *EPSPS*, and *α-tubulin* resistance mutations (Yu *et al.*, 2013; Han *et al.*, 2016; Chen *et al.*, 2019). This makes early detection of the mutation/resistance in the field challenging.

Although enabling rice plant survival at and above field glufosinate rates, the contribution of the Ser59Gly mutation to glufosinate resistance in the R *E. indica* population may need further quantification by genetic approaches (e.g. gene editing to reverse the mutation to the WT in R plants). As the Ser59Gly mutation alone cannot fully account for the resistance level (up to 20-fold) observed in the R *E. indica* population (Janaludin *et al.*, 2015), other unknown resistance mechanisms, including enhanced antioxidant capacity, may be involved and remain to be determined.

In conclusion, parallel evolution of the GS1 Ser59Gly mutation was revealed in glufosinate-resistant populations from Malaysia and China in the globally important C_4_ weed *E. indica*. The emerging Gly59 resistance allele is still relatively rare in the wild as compared with other herbicide resistance alleles [e.g. acetolactate synthase (ALS), ACCase, or EPSPS]. However, it will be enriched and other stronger mutations will be selected with continued and increasing selection pressure due to increasing glufosinate usage, as learnt from the history of herbicide resistance evolution. This outcome, together with emerging non-target site metabolic resistance alleles (e.g. Brunharo *et al.*, 2019), threatens the sustability of herbicide technology involving glufosinate.

## Supplementary data

The following supplementary data are available at *JXB* online.

Fig. S1. Purification of *E. indica* mutant (EiGS1-1-R59) and wild-type (EiGS1-1-WT) proteins from yeast cells.

Fig. S2. Diagram of molecular interactions of glutamate (A) and glufosinate (B) at the binding site on the surface of *E. indica* GS1-1 WT isoform.

Fig. S3. Diagram showing molecular interactions of Ser59 on the surface of *E. indica* GS1-1 WT (A) and mutant (B) isoforms.

erac008_suppl_supplementary_figs_S1-S3

## Data Availability

Sequence data have been deposited in GenBank with accession numbers MZ888499, MZ888500, MZ888501, and MZ888502, and plant materials are available from the corresponding author, Qin Yu, upon request.

## Abbreviations

EiGS: *Eelusine indica* GS
GS: glutamine synthetase
