# Supplementary material for: A naturally evolved mutation (Ser59Gly) in glutamine synthetase confers glufosinate resistance in plants
Source: J Exp Bot. 2022 Jan 13;73(7):2251–62. doi: 10.1093/jxb/erac008 (PMC8982429; doi:10.1093/jxb/erac008)
Supplement: erac008_suppl_supplementary_figs_S1-S3 [file erac008_suppl_supplementary_figs_s1-s3.pdf]

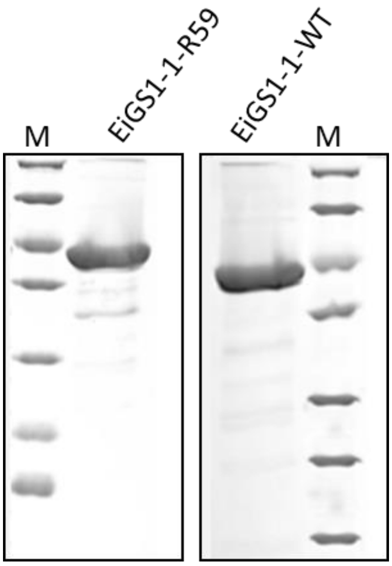

Figure S1. Purification of *E. indica* mutant (EiGS1-1-R59) and wildtype (EiGS1-1-WT) proteins from yeast cells. M refers to protein size markers (from the top to the bottom: 116, 66, 45, 35, 25, 18.4 and 14.4 kDa)

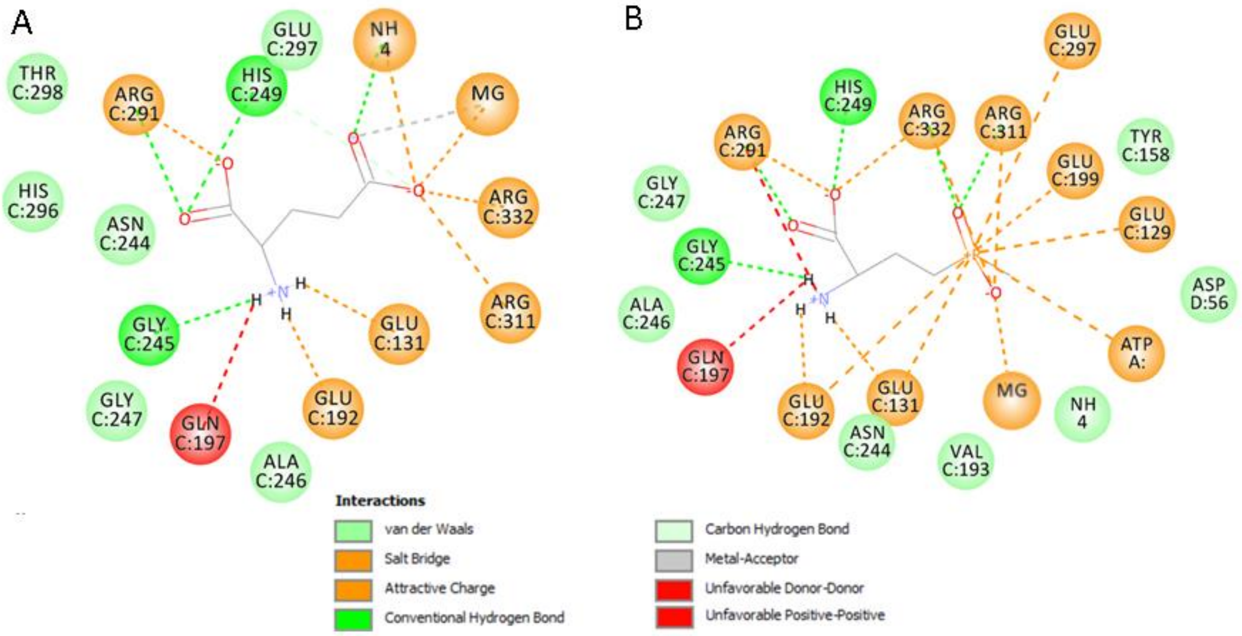

Figure S2. Diagram of molecular interactions of glutamate (A) and glufosinate (B) at the binding site on the surface of *E. indica* GS1-1 WT isoform. Some interactions were hidden. Residues of different GS1 subunits were labeled by letters C and D.

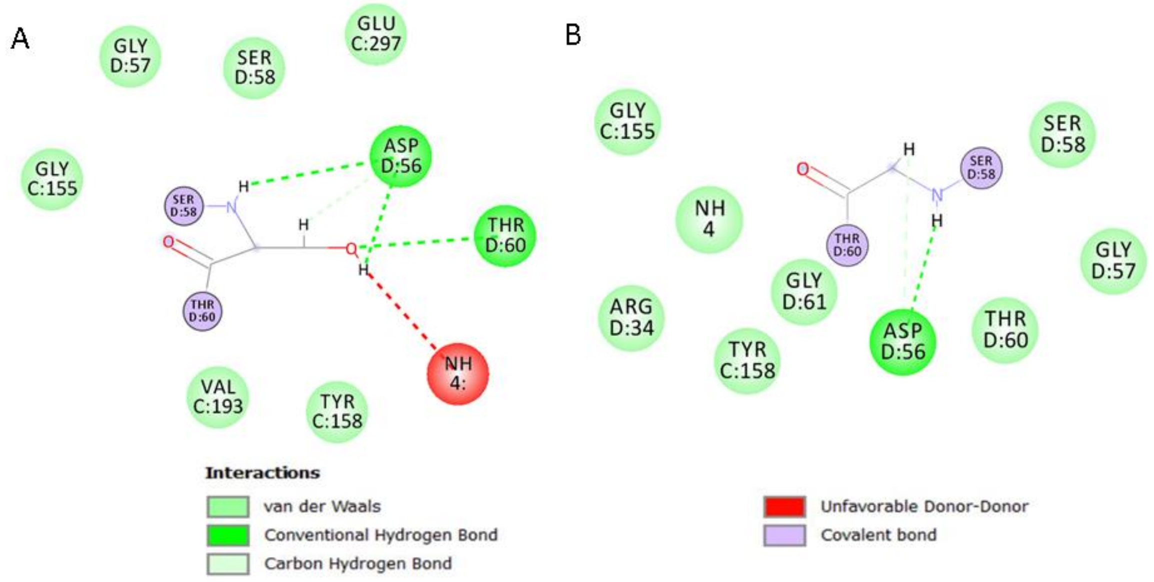

Figure S3. Diagram showing molecular interactions of Ser-59 on the surface of *E. indica* GS1-1 WT (A) and mutant (B) isoforms. Residues in position 59 were shown by sticks. Residues of different GS1 subunits were labeled by letters C and D.
